# Nanofiber Scaffolds as Drug Delivery Systems to Bridge Spinal Cord Injury

**DOI:** 10.3390/ph10030063

**Published:** 2017-07-05

**Authors:** Angela Faccendini, Barbara Vigani, Silvia Rossi, Giuseppina Sandri, Maria Cristina Bonferoni, Carla Marcella Caramella, Franca Ferrari

**Affiliations:** Department of Drug Sciences, University of Pavia, Viale Taramelli, 12, 27100 Pavia, Italy; angela.faccendini01@universitadipavia.it (A.F.); bara.vigani@unipv.it (B.V.); giuseppina.sandri@unipv.it (G.S.); cbonferoni@unipv.it (M.C.B.); carla.caramella@unipv.it (C.M.C.); franca.ferrari@unipv.it (F.F.)

**Keywords:** spinal cord injury, nanofibers, electrospinning, neuroprotection, neuroregeneration

## Abstract

The complex pathophysiology of spinal cord injury (SCI) may explain the current lack of an effective therapeutic approach for the regeneration of damaged neuronal cells and the recovery of motor functions. A primary mechanical injury in the spinal cord triggers a cascade of secondary events, which are involved in SCI instauration and progression. The aim of the present review is to provide an overview of the therapeutic neuro-protective and neuro-regenerative approaches, which involve the use of nanofibers as local drug delivery systems. Drugs released by nanofibers aim at preventing the cascade of secondary damage (neuro-protection), whereas nanofibrous structures are intended to re-establish neuronal connectivity through axonal sprouting (neuro-regeneration) promotion, in order to achieve a rapid functional recovery of spinal cord.

## 1. Introduction

Spinal Cord Injury (SCI) results in devastating and debilitating conditions such as severe dysfunctions of the motor, sensory, and autonomic systems [1].

**Figure 1:**
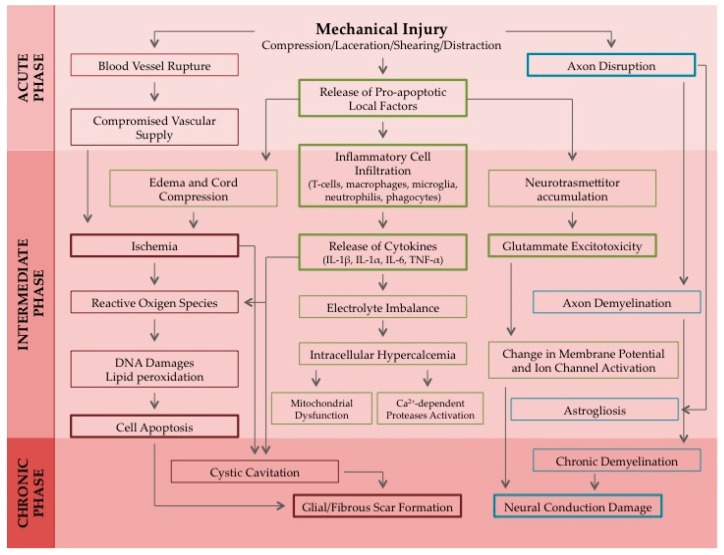
Schematic representation of the pathophysiological response to a spinal cord injury induced by a mechanical trauma. A cascade of vascular, cellular, and biochemical events brings to the progression of the spinal cord damage until the formation of a glial scar. Acronyms: IL-1α, Interleukin 1α; IL-1β, Interleukin 1β; IL-6, Interleukin 6; TNF-α, Tumor Necrosis Factor α.

It is mainly caused by mechanical trauma of the spine, due to traffic accidents, falling from buildings, gun shots, sport injuries etc. It is considered a global issue, affecting people of every age and concerning almost 2.5 million patients worldwide. Despite the fact that, in the last decades, improvements in medical care have increased patient survival rates and reduced the impact of SCI on life quality, a therapeutic approach for the regeneration of damaged cells and for the recovery of motor functions is still lacking [2]. Therefore, effective multifaceted therapies, aiming at reducing the extent of tissue disruption and improving neurologic outgrowth after spinal cord trauma, are urgently needed. The pathophysiological response to a spinal cord injury involves a primary damage, followed by the activation of a cascade of secondary events. SCI instauration and progression are generally divided into acute (seconds to minutes after the traumatic event), intermediate (minutes to weeks after the trauma) and chronic (months to years) phases [2,3] (Figure 1).

Primary mechanical trauma of the spinal cord, such as compression and shear forces, produces instantaneous vascular, cellular, and axonal damages that expand from the injury site in both radial and axial directions [1,4,5].

Vertebral disc fractures produce bone fragmentation and consequent disconnection of long axonal tracts, thus damaging neurons, oligodendrocytes, and astrocytes. After capillary rupture at the injury site, an invasion of blood circulating leukocytes occurs. Such an event, in conjunction with the release of several substances from the damaged cells, determines a toxic environment that is responsible for lesion expansion (SCI secondary phase). Fibroblasts infiltrate the perilesional region while the surviving astrocytes release growth inhibitory chontroitin sulfate proteoglycans (CSPGs), such as neurocan, versican, brevican, phosphacan, as chemical barrier [6,7].

Axon distal segment, unconnected with the neuron soma, crushes and activates a process called Wallerian degeneration, which determines apoptosis of the oligodendrocytes surrounding distal segments [2,8]. The secondary damage ends with the formation of a glial scar, which can further impede axonal regeneration.

Current treatments of SCI can be classified as neuro-protective or neuro-regenerative ones. Neuro-protective therapies are intended to avoid or prevent further progression of the secondary injury; neuro-regenerative approaches are directed to recover the lost or impaired functionality of the spinal cord by repairing the broken neuronal circuitry [9,10].

The aim of the present review is to provide an overview of the therapeutic neuro-protective and neuro-regenerative approaches for SCI treatment. At first, a panorama of current pharmacological approaches is presented with particular regard to drugs whose potential in SCI treatment has been reported in the literature of the last two years. The second part of the review is devoted to emerging technological approaches based on nanofibers. They act as drug carriers, releasing the active compound/saccording to the therapeutic requirements, and provide a physical support, directing and guiding axonal regeneration. Drugs released by nanofibers aim at preventing the cascade of secondary damage (neuro-protection), whereas nanofibrous structures are intended to re-establish neuronal connectivity through axonal sprouting (neuro-regeneration) promotion, in order to obtain a rapid functional recovery of the spinal cord.

## 2. Overview of Current Pharmacological Approaches for the Treatment of SCI

Current pharmaceutical strategies for the treatment of SCI are intended to avoid or prevent secondary injury progression, by minimizing apoptosis, oxidative stress or inflammation [11].

Nowadays, the glucocorticoid Methylprednisolone (MP) is the only drug approved by the Food and Drug Administration (FDA) for SCI treatment. While it determines neuro-protection in the early 8 h after injury, thanks to the reduction of secondary inflammation and lipid peroxidation, it is unable to effectively prevent or stop damage progression. The use of MP is controversial due to its severe complications like pneumonia, sepsis and death [1,12,13]. In addition to MP, in recent years other drugs have obtained promising results when tested in preclinical studies. They were recently reviewed by Kabu et al. [1]. Table 1 reports a list of such drugs together with their mechanism of action and potential effect on SCI. Among them, Riluzole and Minocycline are the most promising.

In the last two years, many research studies, focusing on the identification of drugs potentially effective in SCI treatment, have been published [32,33,34,35,36,37,38,39,40,41,42,43,44,45,46,47,48,49,50,51,52,53,54,55,56,57,58,59,60,61,62,63,64,65,66,67,68,69,70,71,72,73,74,75,76,77,78,79,80,81,82,83,84,85,86,87,88,89,90,91,92,93,94,95,96]; these drugs are listed in Table 2, Table 3, Table 4 and Table 5. Information about mechanism of action, potential effect on SCI and administration route, when mentioned, is reported. In particular, some bioactive vegetal extracts and compounds have been proposed as reported in Table 2. Among them, Ganoderma Lucidum (GL) seems the most promising. Histopathological evaluation and neurological examination demonstrated that GL polysaccharides have in the SCI animal model an effect comparable to that obtained in an animal control group treated with intraperitoneal injection of MP [44]. Also Caffeic acid and Mangiferin show comparable in vivo effects to MP [36,48].

Table 3 lists synthetic drugs having neuro-protective or neuro-regenerative actions, studied over the last year. Among them, Tamoxifen is of particular interest. It is an FDA approved selective estrogen receptor modulator with several neuro-protective properties. Some authors demonstrated its capability to improve functional locomotion recovery after SCI. Results suggested that the mechanism of action of Tamoxifen is to modulate antioxidant, anti-inflammatory, and anti-gliotic responses. Sex differences in response to Tamoxifen and the administration therapeutic window are still unknown [81]. Another drug that could be easily translatable in clinical trials, since it is safe for humans, is Acetyl-l-carnitine. It has a specific action mechanism, improving mitochondrial respiration in the animal model [51]. The effect of drug combination was also investigated. Recently the association of MP with Rosiglitazione showed an increase in functional recovery with respect to that observed when drugs were administrated alone [77]. Promising results were obtained for the Buspirone/Levodopa/Carbidopa combination (Spinalon™) in a double-blind randomized study of phase I/IIa involving patients with a complete spinal cord injury according to the American Spinal Injury Association Impairment Scale (AIS) [83]. Analgesic drugs with a neuropathic-pain target are shown in Table 4, whereas those effective in neurogenic detrusor activation are listed in Table 5. Such drugs are needed since SCI is frequently responsible for a disorder of the normal function of the lower urinary tract, the storage and evacuation of urine. In particular neurogenic detrusor overactivity (NDO) is characterized by a reduced bladder capacity, an elevated detrusor pressure during the storage phase and/or a reduced compliance. NDO can cause an irreversible deterioration of the upper urinary tract with subsequent renal failure [94].

## 3. Overview of Promising Nanotechnology Approaches for the Treatment of SCI

In the context of SCI, an effective drug dose has to be administered in order to reach the injury site and achieve the therapeutic effect. In the case of systemic administration, drugs have to cross the blood–spinal cord barrier (BSCB) to reach the injury site [97]. Oral administration undoubtedly encounters higher patient compliance than parenteral one, but limited gut absorption and/or high pre-systemic metabolism could determine a poor drug bioavailability. Drug administration via epidural and intraspinal routes could be more targeted and effective than systemic delivery, even though several limitations have to be considered. Drugs, administered via the epidural route, have to cross meninges (dura, arachnoid and pia); the use of an intrathecal catheter or repeated spinal injections can represent a high risk of infections [97]. Implanted drug delivery systems have recently emerged as a promising strategy to fill the site of injury. Recently, locally administered drug delivery carriers, such as nanofibers have been proposed in the literature. In vivo tests demonstrated that drug-loaded nanofibers show enhanced therapeutic effects as well as great potential for clinical use. As already mentioned, these systems provide a physical support, directing and guiding axonal regeneration, and, at the same time, modulating the release of the loaded drug/s in response to the therapeutic requirements [2,97].

The capability of polymer-based nanofibers to promote nerve regeneration acting as a support for cell growth and new tissue formation has been widely studied [98,99,100]. Guo et al. reviewed nanofiber scaffolds used for SCI treatment, focusing on peculiar properties such as supporting graft cells, reconstructing tissue loss, alleviating inflammation, improving axonal regeneration, and acting as drug delivery systems [101].

In a more recent work, Asghari et al. explained in detail the properties that a scaffold based on synthetic or natural biodegradable polymers must have in order to be used in tissue engineering [102].

Scaffold must be able to mimic the fascicular nerve architecture and the fibrous extracellular matrix (ECM) that characterized the native tissue in terms of both chemical composition and physical structure [103]. ECM has a complex composition: it contains proteoglycans, proteins, and signaling molecules. It is known for its role in providing structural support to cells and as a location for cells migration [104].

Aligned nanofibers are able to mimic the oriented fascicular nerve environment; in the literature, several studies demonstrated that neurites preferred aligned oriented fibers than randomly oriented ones [105]. Two explanations could clarify such cell behavior. The first one is supported by the experimental evaluation of cell growth: in particular C17.2 and PC12 neural cells were characterized by a significantly higher proliferation when cultured on aligned oriented fibers in comparison with randomly un-oriented fibers [106,107,108]. The second one is based on the reasonable hypothesis that nerve cell outgrowth does not meet any barrier to graft the aligned fibers in comparison with the randomly oriented ones [106]. Zuidema et al. demonstrated that fiber misalignment can significantly impede astrocytes migration and elongation [108].

Different categories of biomaterials were investigated for their ability to guide axonal regeneration and to deliver small molecules at the site of injury or to improve the viability of transplanted stem cells. Appropriate scaffolds for tissue engineering applications should be biocompatible, non-toxic, non-mutagenic, and non-immunogenic. Furthermore, they should be able to provide appropriate mechanical support and show favorable topographical properties to improve cell adhesion, proliferation, and differentiation [109].

The materials mainly used for nanofiber scaffolds used for SCI treatment are completely biocompatible and biodegradable to avoid a second surgical treatment to remove the implanted fibers. The nerve matrix conduits, which are Food and Drug Administration (FDA) and European Commission approved, consist of biodegradable materials, among them the Neurotube™, Neura-Gen™ and Neurolac tubes, are made of poly(glycolide) (PGA), collagen and poly(DL-lactide-ε-caprolactone), respectively.

As listed in Table 6, the common biodegradable synthetic polymers tested in therapy of SCI include: polycaprolactone (PCL), polyethylene glycol (PEG), poly(lactic acid) (PLA), poly-L-lactic acid (PLLA), poly(lactic-co-glycolic acid) (PLGA), silica (SNF), poly-d-lysine (PDL), aminopropyl-trimethoxysilane (APTS), peptide anphiphile (PA) and poly-propylene carbonate (PPC). Natural polysaccharides, such as chitosan and tragacanth gum, and proteins, such as collagen and silk fibroin (SF), are also widely used. Synthetic polymers can provide sufficient mechanical properties and improved manufacturing process, while natural polymers can improve cell attachment and orientation, mimicking the axonal environment [109].

Many authors proved that nanofiber scaffolds strongly improve axonal regeneration in chronic spinal cord injury [115,120,121,133,134,135,136,137].

So far, only a few studies have proposed a combined therapeutic approach, ensuring the regeneration of injured spinal cord by implanting suitable biocompatible scaffolds and by modulating secondary damage response by locally administration of neuro-protective agents. The development of drug delivery nanosystems having both neuro-protective and neuro-regenerative effect is still a challenge.

In the following paragraphs, an overview of the electrospun nanofibers proposed in recent years as drug carriers for the treatment of SCI is given. Particular attention is devoted to manufacturing strategies adopted to achieve optimal drug loading and release.

**Figure 2:**
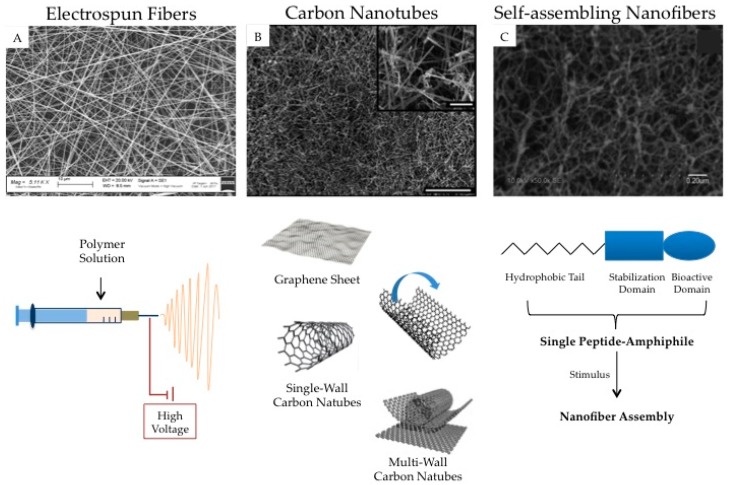
Nanotechnological approaches for the fabrication of fibrillar structures for the treatment of SCI. (**A**) Scanning electron micrograph (Zeiss EVO MA10 (Carl Zeiss, Oberkochen, Germany) shows random dextran/alginate fibers; (**B**) Scanning electron micrograph of carbon nanotubes; scale bars: 250 and 25 μm (inset) (adapted [138]); (**C**) Scanning electron micrograph of self-assembling nanofibers (adapted from [139]).

Carbon nanotubes and self-assembling nanofibers represent other interesting nanotechnology based-approach proposed for SCI treatment. A brief summary of the most meaningful experimental findings on these topics is given. The possibility of using nanostructures as cell carriers is also considered. In Figure 2, a schematic representation of electrospun nanofibers, carbon nanotubes, and self-assembling nanofibers is reported.

### 3.1. Electrospun Nanofibers

**Figure 3:**
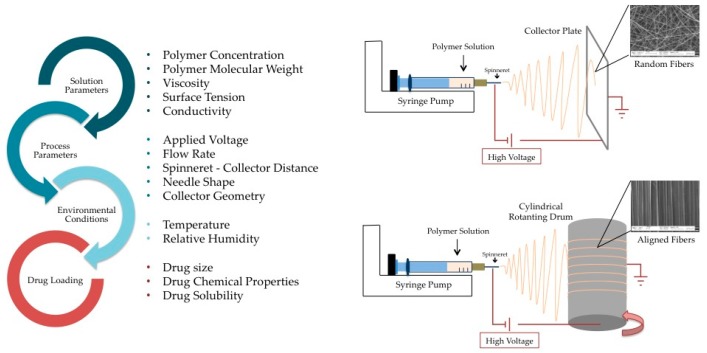
Electrospinning process. On the left, the parameters influencing fiber size, morphology, and density are listed; the physicochemical properties of the loaded drugs are also to be considered when the electrospinning technique is used for the fabrication of drug delivery systems. On the right, a schematic representation of the electrospinning apparatus with particular attention on the collector geometry, which is a crucial variable affecting fiber alignment. Scanning electron micrographs (Zeiss EVO MA10 (Carl Zeiss, Oberkochen, Germany)) show random dextran/alginate fibers and aligned polyethilenoxide/alginate ones.

Electrospinning is of great interest in nanofiber manufacturing due to its simplicity, low cost and great versatility for drug delivery [140]. Figure 3 reports the solution, process, and environmental parameters, which can influence the morphology, size, and density of the electrospun product [140,141] Moreover, a schematic representation of the electrospinning apparatus and process is also provided.

The drug release profile can be modulated by using appropriate polymers and different electrospinning techniques, as hereafter described.

#### 3.1.1. Solution Electrospinning

One-phase electrospinning technique implies that the drug is dispersed or dissolved in a polymer solution and subsequently electrospun. Experimental evidence indicated that blending a hydrophilic drug with hydrophilic polymers (i.e., PEG, PLA, PLGA, polysaccharides, collagen etc.) instead of hydrophobic ones (i.e., PCL) greatly improves drug-loading efficiency by promoting a homogeneous dispersion into the fiber matrix, avoiding the burst effect induced by an excess of drug close to the nanofiber surface [142,143,144]. Drug affinity for the selected polymeric blend should avoid drug molecule transfer towards the nanofiber surface upon storage [60].

Polymer modification and copolymerization represent optimal strategies to control the bio-erosion of the nanofiber carriers due to biological fluids [145,146,147].

Recently, Pires et al. loaded Ibuprofen (a hydrophobic anti-inflammatory drug) into electrospun fibers based on poly(trimethylenecarbonate-co-ε-caprolactone) (p(TMC-CL), by dissolving both the drug and the copolymer in a mixture of dichloromethane (DMC) and dimethyl formamide (DMF) [122]. This study demonstrated that the solvent mixture composition influences fiber morphology and diameter; in particular fiber diameter reduction was observed on increasing DMF content. Depending on the solvent used, different release mechanisms were observed. Drug release was diffusion-dependent for fibers prepared from DCM solutions, in contrast to fibers prepared from DCM/DMF mixtures where a burst release occurred. The results evidenced that the selection of an appropriate solvent or solvent mixture for drug and polymer dissolution can represent a good strategy to modulate drug release from nanofibers [122].

Recently, some interesting approaches were developed for hydrophobic drug loading into hydrophobic polymer nanofibers. In particular, Hsu et al. developed a PCL-based hybrid drug release system, consisting of nanofibers and microbeads for a month-long release of Dexamethasone (DXM) [119]. The authors evidenced that it was possible to achieve an extended release of DXM by the combination of increased crystallinity of the electrospun mats with a hybrid structure of 1.5–4 μm diameter beads and nanofibers. Such an approach may find application for neuro-regenerative drug delivery.

#### 3.1.2. Emulsion Electrospinning

When a drug is insoluble in the polymer solution, it can be incorporated within the fiber structure using a process known as emulsion electrospinning [145]. A drug aqueous solution is mixed with a hydrophobic polymer solution, defined as the oily phase. After electrospinning, the drug-loaded aqueous phase is dispersed within the nanofiber matrix; core-shell nanofibers can be obtained when the polymer is also dispersed in the aqueous phase [148]. The main advantage of such a technique is to avoid drug stability problems possibly due to polymer and drug dissolution in respective suitable solvents. Emulsion electrospinnig provides the chemical separation of drug and polymer by employing a single vehicle instead of two different solutions as in the case of coaxial electrospinning [109].

In comparison with the coaxial electrospinning, the emulsion technique could determine degradation of unstable macromolecules, due to the interfacial tensions at the organic/aqueous interface of the emulsion. Several problems may be encountered, for example, with proteins due to their size and three-dimensional structure [146,148,149,150].

#### 3.1.3. Coaxial Electrospinning

Coaxial electrospinning differs significantly from the emulsion method since the core-shell fibers are manufactured starting from two solutions and using two electrospinning tips [149,150]. Core-shell fibers exhibit more advantages than monolithic ones, such as modulation of drug release mechanism and kinetics via control of shell properties (i.e., thickness, porosity, biodegradation), versatility in drug selection, and preparation of multifunctional fibers (i.e., fibers characterized by a core controlling drug release and a shell improving cell adhesion). Coaxial electrospinning disadvantages are related to solvent evaporation and to the difficulty of simultaneously electrospinning two different polymer solutions with peculiar electrodynamic behavior. Since fiber morphology (core-shell structure) could prevent rapid solvent evaporation, the residual amount of organic solvents remaining in the fibers has to be detected. Due to the electrospinning process complexity, rheological and interfacial properties of the two polymer solutions as well as spinning parameters (applied voltage, spinneret-collector distance and flow rate) have to be carefully chosen [146,149,150,151].

Recently, coaxial nanofibers have been extensively investigated as biomolecule (proteins, growth factors) carriers or cell delivery scaffolds for the treatment of SCI, but only a few papers have been published on the delivery of low molecular weight drugs as nerve protective agents. Coaxial nanofibers are generally characterized by an initial burst release followed by a controlled one [146]. The initial burst effect, caused by the presence of drug molecules on fiber surface, is functional to the treatment of primary spinal cord injury, contributing to reduce even the cascade of secondary events. The subsequent prolonged drug release, guaranteed by the drug loaded in the fiber core, is useful to slow down SCI progression, particularly in the chronic phase; drug release is controlled by drug diffusion across the polymer matrix and system slow biodegradation. Different drugs can be encapsulated in both the fiber shell and core, achieving a binary release.

Nanofiber-based scaffolds, characterized by the shell and core with a peculiar morphology, have been developed also to improve the system interaction with nerve cells. In particular, Zamani et al. designed PLGA electrospun fibrous scaffolds with a nano-rough sheath and an aligned core [98]. They manufactured a three-dimensional nanofibrous scaffold by a combined electrospinning method with a water vortex and a two-nozzle system. The authors studied nerve cell morphology and proliferation in the developed scaffold. Thanks to scaffold nano-structure, nerve cells strongly attached on the fiber nanoporous shell, penetrated the inner structure and orientated along the aligned fiber direction of the core. These scaffolds were shown to support axonal regeneration of injured spinal cord in a rat model [98].

#### 3.1.4. Drug Loading

A strategy proposed in the literature to avoid drug denaturation during fiber manufacturing or to overcome low drug solubility in polymer solution is physical drug adsorption on the fiber surface. This approach generally determines a burst release of drug that could be useful, as already mentioned, for the treatment of primary SCI. Burst release can be avoided by drug bonding to the fiber surface via covalent coupling (drug-conjugated nanofibers) [2,148,151].

Recently, Schaub et al. suggested that surface modifications may be determined through transient covalent bonds, lasting less than a few days [152]. In this study diethylenetriamine (DTA) and 2-(2-aminoethoxy)ethanol (AEO) were covalently attached to the surface of an electrospun fiber-based on PLLA. Such surface modifications improved scaffold hydrophilicity, but surprisingly no differences were observed between the modified fibrous system and the unmodified control one in terms of neurite extension. The authors evidenced that both AEO and DTA were rapidly removed from the scaffold surface [152].

Raspa et al. blended PCL and PLGA with biologically active peptide sequences (SAPs), modifying the surface properties of the electrospun scaffolds in order to improve nerve regeneration [110]. SAPs were immobilized for a longer time and nanofibers provided support for cell growth. Two systems consisting of SAPs encapsulated in PCL–PLGA coaxial electrospun scaffolds were investigated: the first was composed of a core of self-assembling peptides (SAP AC-FAQ) and by a PCL–PLGA-based shell, whereas the second one was characterized by a core containing a PCL–PLGA emulsion and a shell based on an emulsion of PCL–PLGA and a functionalized SAP AC-FAQ. The second one was characterized by the best performance in terms of cell viability and tissue response [110].

Besides maintaining drug activity, it is important to investigate if drug incorporation into nanofibers modifies fiber morphology. In fact, it has been recognized that fiber diameter could affect cellular functionality; in particular, cells adhere and expand mainly on fibers with a diameter close to the cell dimension [2]. The change in fiber diameter due to drug loading can moreover interfere with a correct interpretation of nanofiber performance: in fact, both the changes in fiber diameter and drug release can be responsible for the effects on neurite extension. Schaub and Gilbert observed a decrease of fiber diameter due to incorporation of the antimetabolite 6-Aminonicotinamide (6AN) [153]. Such a result can be explained by the impediment of polymer chain entanglement during electrospinning due to charged drug molecules. The reduction of fiber diameter is generally followed by a decrease of fiber alignment [2].

Johnson et al. proved that the inclusion of either Riluzole or neurotrophin-3(NT-3) into electrospun PLLA fibers via emulsion electrospinning had significant effects on fiber physical characteristics, in particular determined a decrease of both fiber diameter and alignment [2].

One interesting work from Pires et al. demonstrated that the polarity of the polymer solution could affect the diameter of the resulting electrospun fibers [154].

Moreover, the solid state of the drug is an important parameter that can significantly affect drug distribution and release kinetics as well as nanofiber physical stability upon storage. Seif et al. investigated the formation of Caffeine (chosen as hydrophilic model drug) crystals in electrospun fibers when two different polymers were used: the hydrophilic poly(vinyl alcohol) (PVA) and the hydrophobic PCL [155]. They proved that solvent polarity has the major effect on crystal formation, whereas a minor effect derives from the electrospinning process parameters. Therefore, uncontrolled drug crystallization can be prevented and controlled drug delivery from electrospun fibers can be achieved by adjusting the polarity of the solvent mixture and optimizing the process parameters [155].

#### 3.1.5. Drug Release

One fundamental issue concerning material selection in the design of implantable scaffolds for the treatment of nerve regeneration is the rate of biodegradation or bioerosion. The terms biodegradable and bioerodible are frequently used as synonyms, but they represent two different concepts. Biodegradable polymers are materials able to disassemble In vivo, by forming small fragments that move away from the wound site; the term bioerodible refers instead to those polymers that are subjected to In vivo degradation, with complete elimination of the starting material [109]. Depending on the material employed, the release of the drug loaded into the implantable scaffolds can occur according to specific mechanisms and kinetics: diffusion through the polymer network or matrix pores and polymer degradation or erosion.

Slowly bioerodible scaffolds loaded with small hydrophilic molecules are reported as an example. After implantation, the initial rate of drug release will exclusively depend on drug diffusion and not on scaffold erosion, which becomes important at a later time. Conversely, if the matrix biodegradation or bioerosion rate is higher than the drug diffusion, drug release will be controlled by polymer erosion or the biodegradation process. For these reasons, slowly degrading polymers are commonly used as a support for nerve guidance in SCI animal models, considering that a period of 3–6 months is required to achieve a functional regeneration [136].

Drug release from implantable polymer-based scaffolds, such as electrospun fibers, could be moreover influenced by scaffold composition; therefore, the hydrophilicity and molecular weight of the selected polymers are crucial parameters to be considered. Cross-linking of polymer chains could be also induced to control drug diffusion, as long as nanofibers act as support of nerve outgrowth [136].

One of the first small organic molecules released from electrospun fibers intended for spinal cord repair was the antimetabolite 6-Amino-nicotinamide (6AN). This is known to inhibit astrocyte metabolism at low levels, and to exert a lower effect on neurons. In particular, Schaub and Gilbert developed 6AN loaded emulsion–electrospun PLLA nanofibers [153]. 6AN was found to inhibit astrocyte viability without interfering with neurite extension. The inhibition of astrocyte viability should reduce the negative effects due to astrocyte reactivity after SCI, but no in vivo experiments were affected [153].

A more in-depth study was carried out on Rolipram by Downing et al. [156]. Rolipram was loaded into electrospun PLLA nanofibers and implanted in a SCI rat model. The authors demonstrated that animals treated with electrospun nanofibers, able to release low drug doses of (~3 μg/cm^2^ over 12 days), presented significantly improved functional recovery after injury in comparison with an untreated group (controls). Interestingly, the group of animals treated with electrospun fibers releasing a low Rolipram dose showed a significantly improved motor function with respect to another group treated with electrospun fibers releasing a large amount of Rolipram (~60 μg/cm^2^ over 12 days). This study, even if not recent, focused on the capability of nanofibers to load and release therapeutic levels of drug for SCI treatment [156].

### 3.2. Carbon Nanotubes

A particular type of nanofibers is represented by carbon nanotubes (CNTs), which are composed of graphene sheets rolled up to form a cylinder made of carbon atoms. CNTs are classified according to the number of layers: single-walled (SWCNTs), double-walled (DWCNTs), and multi-walled (MWCNTs) carbon nanotubes. They are characterized by large specific surface area, electrically conductive, elastic resistance, and nanostructure mimicking the extracellular matrix (ECM). These properties make them good candidates for nerve tissue regeneration [157].

Moreover, CNTs are able to regenerate neuronal electrical activity by settling contacts with cell membranes to create electrical ‘short-cuts’ between various areas of the neuron [157,158]. Recently, Palejwala et al. prepared graphene nanoscaffolds by mild chemical reduction of graphene oxide [115]. They were implanted in a rat model immediately after hemispinal spinal cord transection, using a hydrogel matrix as vehicle. A group of animals were treated with the pure hydrogel matrix (controls). The scaffolds proved to be biocompatible and hystological evaluation pointed out the growth of connective tissue elements, blood vessels, neurofilaments, and Schwann cells around the scaffolds [115]. In another study, López-Dolado et al. proved that graphene oxide-based scaffolds promoted, after implantation, the migration of M2 macrophages into the damaged site [159].

### 3.3. Self-Assembling Nanofibers

A recent nanotechnology approach for spinal cord repair is represented by self-assembling (peptide) nanofibers (SAPNs). Such nanofibers are based on the synthesis of unique amphiphilic peptides, which are characterized by the periodic repetition of hydrophobic and hydrophilic aminoacids, alternatively arranged. These peptides spontaneously self-assemble into well-ordered nanofibers after contact with physiological fluids, containing electrolytes and salts. These peptides in solution can be easily injected into the nervous injury and arrange themselves in vivo into a stable nano-fiber 3D-matrix thanks to the ionic strength, pH, and temperature of the physiological environment. They fill up the site of injury without producing secondary damage, as may be the case for other more rigid scaffolds (such as nanofiber implanted system). SAPNs may be built from natural and biocompatible peptides, thus they should be promising biomaterials for regenerative medicine [135,160].

Zhang et al. discovered the sequence AcN-RADARADARADARADA-CNH2, named RADA 16-I, as artificial SAP, which can form a stable nanostructure when in contact with physiological ions [161].

Inspired by the properties of RADA 16-I on cell culture, Guo and coworkers injected the SAP RADA 16-I in a rat spinal cord injury model with very good results (six weeks after transplantation the cavity was filled up by the scaffold) [162].

Subsequently Gelain and coworkers combined microstructured electrospun nanofibers (a mixture of PLGA and PCL) with the nanostructured SAP RADA 16, thus providing *in situ* delivery of cytokines. In such a hybrid scaffold the SAP can self-assemble into the PLGA/PCL electrospun microguidance fibers, presoaked in PBS. Six months after transplantation in animals, conspicuous cord regeneration was still active, accomplishing functional reconstruction of chronic spinal cord injury [121].

Several research groups tested the neuro-regenerative potential of SAP alone or in combination with electrospun nanofibers and many new SAP nanofiber scaffolds were developed [135,160,161,162,163,164,165].

Gelain et al. identified a new SAP named Ac-FAQ (Ac-FAQRVPP-GGG-(LDLK)3-CONH2) which promoted significant locomotion recovery in an animal model after injection [164]. Raspa et al. as already described in Section 3.1.4 assembled the novel AC-FAQ SAP with the coaxially electrospun polymeric fibers [110].

In Table 7 examples of self-assembling peptides proposed in the literature for SCI treatment are reported

It is very important to distinguish a nanofiber scaffold, formed through macromolecular self-assembly, from that prepared by coaxial, blending or emulsion electrospinning methods. The physical and chemical properties of the self-assembling structures (e.g., size, shape, internal order, stability, surface, chemistry) are strictly connected with the molecule characteristics forming the network and with the physiological conditions in which assembly occurs [142,168].

Recently, an increasing interest has been observed towards the use of drugs as possible well-defined nanostructures of various sizes and shapes (e.g., nanofibers). This strategy is based on the self-association capability of some drugs to build stable three-dimensional 3D structures [99].

Ma et al. studied how some small drugs can be employed as building blocks in the construction of 3D-nanostructures. Amphiphilic drugs, possessing hydrophobic and hydrophilic groups, could undergo reversible self-assembly, resulting in the formation of dynamic molecular micelles or even discrete supramolecular nanostructures of well-defined size and shape (such as Methotrexate used in neurodegenerative diseases) [99].

Goswami et al. demonstrated that micelles with nanofiber geometry, formed by conjugation of the hydrophobic Deferasirox (DFX) with cell-penetrating peptides (CPPs), were suitable for the delivery of Curcumin, a hydrophobic and anti-neurodegenerative drug with promising SCI applications [169].

### 3.4. Nanofibers as Cell Carriers

The loss of neuronal tissue at the injury site has made cell transplantation an attractive strategy for SCI treatment. Over the last decades, the use of various cell lines has been proposed due to their action in the regeneration process of nervous tissue [1].

Considering that the functional recovery of damaged nerve cells may be compromised by the hostile environment at the injury site, cell transplantation has to be performed within 7–10 days post-injury [165]. Different administration routes have been proposed: intravenous injection, that requires a great amount of cells to effectively target the injury area; intranasal, generally responsible for a lower therapeutic effect with respect to intrathecal infusion [170]; direct transplantation at the site of injury, with the risk that needle penetration produces secondary nerve damages [171]; subarachnoid route, that avoids needle damages and requires optimized procedures to impede iatrogenic problems [172].

In a recent work Kim et al. demonstrated the efficacy of mesenchymal stem cells (MSCs) transplantation in a rat model, using polymeric scaffolds as cell carrier [112]. Two different scaffolds were considered: one based on PLGA, the other one on chitosan. The control was represented by intralesional injection of MSCs without carrier. Engraftment and differentiation of the transplanted cells, expression of neurotrophic factors in the injured spinal cord and functional recovery, were evaluated for each group. It was demonstrated that the carrier-mediated approach promoted a better cell engraftment and neuroprotection than intralesional injection of cell suspension. The functional improvement was particularly evident in the animal group treated with MSC-chitosan scaffold [112].

In recent years, electrospun nanofibers encapsulating living cells have been proposed as new carriers able to improve tissue regeneration at the implantation site. Such scaffolds represent a solution to the shortage of nerve tissue useful in repair and replacement surgeries, even though the preservation of post-electrospinning viability of cells is still a challenge [172,173].

## 4. Conclusions

Recently, several studies have been carried out to better understand the pathological consequences of spinal cord lesion as well as the advantages and limitations of both neuro-protective and neuro-regenerative interventions. One of the most promising strategies in SCI treatment is the development of therapeutic platforms, able to ensure controlled drug delivery at the site of injury, as well as to promote neurite alignment and outgrowth. Polymer-based nanofibers represent attractive three-dimensional scaffolds for neural regeneration, mimicking the native extracellular matrix and providing topographical cues to axonal regrowth. After implantation, nanofibers should directly interact with resident cells, promoting their attachment, proliferation, and differentiation, thus establishing a pro-regenerative environment at the damaged site. Furthermore, these implanted scaffolds should hydrate upon contact with physiological fluids, forming a matrix characterized by controlled biodegradation which is able to modulate drug release, according to the therapeutic requirements. Concerning this latter aim, the design of multi-layer structures, produced by alternating layers of different polymers, should allow a controlled delivery of the loaded drug.

Currently, a combined approach ensuring both neuro-protective and neuro-regenerative outcomes, thus modulating a plethora of cellular, biochemical and vascular events, could represent a promising strategy for the treatment of spinal cord injuries. Biologists, bioengineers, chemists, pharmacists, and clinical researchers are called to share their skills and knowledge with the aim of developing innovative polymer-based delivery systems. Such systems could be loaded with neuro-protective agents, growth factors, and/or stem cells in order to promote an effective regeneration of damaged neuronal cells and the recovery of motor functions after SCI.

## Figures and Tables

**Table 1:** Drugs reviewed by Kabu et al. [1] as neuro-protective agents for spinal cord injury (SCI) treatment.

Name	Mechanism of Action	Effect on SCI
Atorvastatin (Lipitor) [14]	Reduction of cholesterol levels	Anti-inflammatory effect, anti-apoptosis, tissue sparing and locomotion recovery
Calpain inhibitors [15]	Inhibition of cytoskeletal protein degradation and apoptosis	Tissue preservation, locomotion recovery, anti-apoptosis
Chicago sky blue [16]	Macrophage migration inhibition	White matter increase and blood vessel integrity recovery
Erythropoietin (EPO) [17,18]	Activation of EPO receptor	Anti-inflammatory effect, anti-apoptosis, cytoprotection, vascular integrity recovery, lipid peroxidation inhibition
Estrogen [19]	Hormone replacement	Anti-apoptosis, myeloperoxidase activity reduction, microglial/macrophage accumulation
C3-exoenzyme, Fasudil, Y27532, Ibuprofen [1]	Rho antagonists	Locomotion recovery
Ferulic acid from Ferula species [20]	Antioxidant activity	Anti-inflammatory effect, locomotion recovery, axonal/myelin protection and excitotoxicity prevention
FTY720 [21]	Modulation of sphingosine receptor	Anti-inflammatory effect, anti-apoptosis, tissue sparing and locomotion recovery
Hydralazine [22,23]	Acrolein scavenger	Neuropathic pain reduction and locomotion recovery
Imatinib [24]	Protein-tyrosine kinase inhibitor (clinically used for leukemias and gastrointestinal stromal tumors)	Anti-inflammatory effect, anti-apoptosis, tissue sparing and locomotion recovery
Melatonin [25]	Antioxidant activity	Lipid peroxidation reduction, neuro-axonal and blood-spinal cord barrier (BSCB) protection, locomotion recovery
Minocycline [19]	Antioxidant activity	Immunomodulation of microglia, excitotoxicity, mitochondrial stabilization, anti-apoptosis
NSAIDs [19]	Selective cycloxygenase (COX−2) inhibitors	Anti-inflammatory effect
Quercitin, Deferoxamine and Ceruloplasmin [26,27,28]	Ca^2+^ chelation	Locomotion recovery
Riluzole [29]	Blockage of the sodium channels	Intracellular [Na^+^] and [Ca^2+^] modulation and excitotoxicity reduction
Rolipram [30]	Phosphodiesterase type 4 inhibitor	Anti-inflammatory effect, anti-apoptosis, tissue sparing and locomotion recovery
Vitamins C and E [31]	Antioxidant activity	Anti-inflammatory effect

**Table 2:** Vegetal extract components reported in the literature over the last two years as potentially effective in the treatment of SCI.

Name	Mechanism of Action	Effect on SCI	Administration Route in Animal Models
Allicin [32]	Increase in nuclear factor (erythroid-derived 2)-related Factor-2 (Nrf-2) nuclear translocation in neurons and astrocytes	Neuro-protection, locomotion recovery antioxidant, anti-apoptosis and anti-inflammatory effects	Intraperitoneal injection
Aloe vera [33]	Reduction of neuronal nitric oxide synthase (nNOS) and nuclear factor kappa-light-chain-enhancer of activated B cells (NF-κB) protein	Anti-inflammatory, antioxidant, anti-apoptosis	*Per os*
Asiaticoside [34]	Inhibition of p38-mitogen-activated protein kinase (p38-MAPK) signaling pathway	Antioxidant and anti-inflammatory effects	Intraperitoneal injection
Buyang Huanwu decoction [35]	Reduction in caspase-3 and Bax expression and increase in Bcl-2 expression	Anti-apoptosis effect and hind-limb motor function recovery	*Per os*
Caffeic acid phenethyl ester (CAPE) [36]	Antioxidant activity	Neuro-protection, anti-apoptosis	Intraperitoneal injection
Carnosol [37]	Down-regulation of NF-κB and COX-2 levels and up-regulation of phosphorylated Akt and Nrf-2 expression	Neuro-protection, antioxidant and anti-inflammatory effects	Intraperitoneal injection
Crocin from *Crocus sativus* [38]	Down-regulation of tumor necrosis factor- α (TNF-α) and Interleukin 1β (IL-1β) and antioxidant activity	Neuro-protection and functional recovery in animal SCI	Implantation
Curcumin [39,40]	Reduction of inflammatory cytokine expression and antioxidant activity	Neuro-protection, anti-apoptosis, oxidative stress and lipid peroxidation reduction, locomotion recovery	Intraperitoneal injection
Docosahexaenoic acid (DHA) [41]	miR-21 and phosphorylated Akt up-regulation and phosphatase and tensin homologue (PTEN) down-regulation	Neuroplasticity enhancement	Tail vein injection
(−)-epigallocatechin-3-gallate polyphenol [42]	Down-regulation of Ras homolog gene family, member A (RhoA), fatty acid synthase (FASN) and TNF-α expression	Neuro-protection, reduction of thermal hyperalgesia and of astro- and microglia reactivity	Intraperitoneal injection
Glycyrrhizic acid [43]	Reduction of NF-κB and S100B expression	Neuro-protection, lipid peroxidation reduction, anti-necrotic and anti-inflammatory effects	Catheter inserted into the extradurally thoracic
*Ganoderma lucidum* polysaccharides from Basidiomycota [44]	Modulation of caspase-3 and myeloperoxidase activities, reduction of transforming growth factor- α (TGF-α), malondialdehyde and nitric oxide levels	Neuro-protection and functional recovery	*Per os*
*Ginkgo biloba* extract 761 [45]	Antioxidant, antiapoptosis	Neuro-protection, motor recovery	Intraperitoneal injection
*Go-sha-jinki-Gan* [46]	Anti TNF-α	Neuro-protection, analgesic and anti-necrosis effects	Implantation
*Herba Lycopodii* [47]	Increase of brain derived neurotrophic factor (BDNF) expression	Neuro-protection and motor function improvement	Intragastric injection
Mangiferin [48]	Reduction of malondialdehyde (MDA), superoxide dismutase (SOD), catalase (CAT) activities and serum levels of glutathione peroxidase (GSH-PX), NF-κB, TNF-α, IL-1β, modulation of Bcl-2 and Bax pathway	Neuro-protection, antioxidant and anti-inflammatory effects and anti-apoptosis, locomotion recovery	Intraperitonesl injection
Rutin [49]	Macrophage inflammatory protein-2 (MIP-2) expression inhibition and matrix metalloproteinase-9 (MMP-9) activation, down-regulation of p-Akt expression	Neuro-protection and locomotion recovery	Intraperitoneal injection
Thymoquinone from *Nigella sativa* [50]	Antioxidant activity, modulation of cytokine, activation of antioxidant enzyme	Neuro-protection, antioxidant activity, anti-inflammatory effect, reduction of motor neuron apoptosis	Intraperitoneal injection

**Table 3:** Neuro-protective or neuro-regenerative drugs reported in the literature over the last two years as potentially effective in the treatment of SCI.

Name	Mechanism of Action	Effect on SCI	Administration Route in Animal Models
Acetyl-L-carnitine [51]	Improvement of mitochondria respiration for adenosine tri-phosphate (ATP) production	Protection of endothelial cells of microvessels and locomotor function recovery in lumbar injury	Intrathecal (sub-arachnoid) injection in rats
Adalimumab [52]	Antioxidant, TNF-α, IL-1β and IL-6 serum levels	Neuro-protection and anti-inflammatory effect	Subcutaneous injection in compressive spinal cord injury
Alpha Lipoic Acid + N-Acetyl Cysteine [53]	TNF-α, IL-6 and malondialdehyde (MDA) inhibitor	Motor recovery and anti-inflammatory and antioxidant effects	Intraperitoneal injection
Aspirin [54]	Inhibition of phospholipases, nitric oxide synthetases, and cyclooxygenases	Neuro-protection and, anti-inflammatory effects, lipid peroxidation reduction and locomotion recovery	Intraperitoneal injection
Azithromicyn (AZM, macrolide antibiotic) [55]	Reduction of pro-inflammatory macrophage activation	Anti-inflammatory effect, tissue sparing and motor recovery	*per os*
A68930 (Dopamine D1 receptor agonist) [56]	Inhibition of NLRP3 inflammasome activation and reduction of pro-inflammatory cytokines levels and MPO activity	Neuro-protection and anti-inflammatory effect	Intraperitoneal injection
cAMP combined with functionalized collagen scaffold [57]	Reduction of cavitation volume, axonal and neuronal regeneration	Neuro-regeneration, remyelination, revascularization and locomotion recovery	Implantation
Carvedilol [58]	Increase in SOD and glutathione (GSH), reduction of MPO and malondialdehyde (MDA)	Neuro-protection, antioxidant and anti-apoptosis effects, locomotion recovery	
Dexamethasone [59]	Macrophages modulation	Neuro-protection and locomotor recovery	Subdural infusion
Dibutyryl cyclic adenosine monophosphate (db-cAMP) [60]	Activation of protein kinase A (PKA) signaling by cAMP-related pathways; reduction of apoptosis	Neuro-regeneration, axonal sprouting, functional recovery and modulation of glial scar formation	Implantation
17β-estradiol (E2) [61]	Down-regulation of LC3II and beclin-1 expression and suppression of excessive autophagy	Neuro-protection and locomotion recovery	Intramuscular injection
Estrogen hormone [62]	Reduction of TNF-α and iNOS genes expression	Antioxidant, locomotion recovery and anti-inflammatory effect	Intraperitoneal injection
FK506 (Tacrolimus) + Minocycline [63]	Reduction of thiobarbituric acid–reactive species (TBARS), total glutathione (GSH) and MPO activity	Neuro-protection, functional recovery and antioxidant effect	*Per os*
Gp91ds-tat (NOX2-specific inhibitor) [64]	Inhibition of NADPH oxidase (NOX) enzyme (NOX 2 isoform)	Antioxidant and anti-inflammatory effects	Intrathecal injection
Histamine H4 receptor agonist [65]	Reduction of IL-1β, TNF-α, 8-hydroxy-2′-deoxyguanosine (8-OHdG) and PARP expression and restoration of MnSOD enzymatic activity	Antioxidant, anti-inflammatory and analgesic effects	*Per os*
Histidine-Tryptophan-Ketoglutarate (HTK) solution [66]	Metabolic regulation and blood-flow maintenance agents	Locomotion recovery, neuro-protection and reduction of ischemia	Infusion into the occluded aortic segment
Lipoxin A4 (LXA4) [67]	Reduction of spinal expression levels of microglial markers (IBA-1) and pro-inflammatory cytokines (TNF-α)	Neuro-protection, analgesic and anti-inflammatory effects	Intrathecal injection
Melatonin with amniotic epithelial cells (AECs) [68]	Melatonin receptor 1 stimulation and promotion of ARC differentiation into neural cells by Wint-4 gene expression	Neuro-regeneration and locomotion recovery	Injection along the midline of spinal cord
Metformin [69]	Reduction of NF-κB expression and caspase 3 activation, autophagy activation via mTOR/p70S6K signaling	Neuro-protection, anti-apoptosis and anti-inflammatory effects in preconditioning treatment	Intraperitoneal injection
*N*-(4-cyanophenylmethy)-4-(2-diphenyl)-1-piperazinehexanamide (LP-211) [70]	Serotonin (5-HT7) selective agonism, hyponatremia, hyperkalemia and hypermagnesemia induction	Modulation of imbalances in serum electrolyte concentration, neuro- and renal tissue protection	Intraperitoneal injection
Nor-Binaltorphimine (norBNI) [71]	κ-opioid receptor (KOR) antagonism and morphine antagonism	Locomotion recovery	Intraperitoneal injection
PMX53 (C5aR antagonist) [72]	Inhibition of neutrophil infiltration and reduction of MPO activity	Neuro-protection from ischemia-reperfusion injury	Femoral vein injection
Progesterone [73]	Modulation of pro-inflammatory cytokine expression	Anti-inflammatory, remyelinating action, and analgesic effects	Subcutaneous injection
Propofol [74]	Reduction of superoxide dismutase 1 (SOD1) expression related to PI3K/AKT signal pathway	Reduction of spinal cord ischemia/reperfusion injury and antioxidant effect	intraperitoneal injection in rabbit with ischemia/reperfusion (I/R) spinal cord injury by aortic occlusion
Rapamycin [75]	Activation of Wnt/β-catenin pathway	Neuro-protection and locomotion recovery	Intraperitoneal injection
Retinoic acid (Vitamin A) [76]	Autophagic flux activation after trauma	Neuro-protection, functional recovery and prevention of BSCB disruption	Intraperitoneal injection
Rosiglitazone in combination with MP [77]	Peroxisome proliferator-activated receptor-γ (PPAR-γ) activation	Functional recovery, anti-inflammatory antioxidant and anti-apoptosis effects	Intraperitoneal injection
Selenium-enriched supplement (SES) [78]	Up-regulation of ciliary neurotrophic factor (CNTF) and CNTF-Rα expression	Neuro-protection	*Per os*
Simvastatin [79]	Autophagy activation by mTOR signaling pathway inhibition	Neuro-protection	
Stat 1 Inhibitor (S1491) [80]		Neuro-protection and anti-apoptosis effect	Intraperitoneal injection
Tamoxifen [81]	Estrogen receptor modulator	Anti-apoptotic, antioxidant, anti-inflammatory, anti barrier permeability and antigliotic effects	
Tetramethylpyrazine (TMP) [82]	Activation of Akt/Nrf-2/HO-1 signaling pathway	Neuro-protection, locomotion recovery and reduction of BSCB permeability	Intraperitoneal injection

**Table 4:** Drugs with a neuropathic-pain target, reported in the literature over the last two years as potentially effective in the treatment of SCI.

Name	Mechanism of Action	Effect in SCI	Administration Route
Acrolein [84]	Activation of transient receptor protein ankyrin 1 (TRPA1) in both central and peripheral systems	Reduction of both acute and chronic neuropathic pain	Injection in spinal cord
Botulinum Toxin type A (BTX-A) [85]	Inhibition of the release of substance P, calcitonin and glutamate	Reduction of chronic neuropathic pain	Subcutaneous injection
Cannabis [86]		Reduction of neuropathic pain	Vaporization
GABAergic inhibitors [87]	Reduced neuronal activity in the GABAergic ZI (*zona incerta*)	Reduction of neuropathic pain	Cannula implantation
Methadone [88]	Opioid agonist	Reduction of neuropathic pain during opioid rotation for chronic pain	
Morphine [89]	Toll like receptor 4 (TLR4) pathway attivation and allodynia increase shortly after trauma	Prevention of amplified allodyna in a long/term administration	Subcutaneous injection
Neurothensin A analogue (CGX-1160) [90]		Reduction of neuropathic pain	Intrathecal injection

**Table 5:** Drugs activating the neurogenic detrusor in subject with SCI, reported in the literature over the last two years.

Name	Mechanism of Action	Effect in SCI	Administration Route
Botulinum toxin A [91]		Upper urinary tract protection, modulation of detrusor overactivity and detrusor external sphincter dyssynergia	Injections into detrusor and external urethral sphincter in humans with suprasacral and sacral injuries
Imidafenacin [92]	Anticholinergics selective for the urinary bladder, detrusor pressure reduction and cystometric volume increase	Urodynamic effects with possibly alleviation of bladder complication	Injections in patients with SCI and low cystometric volume and/or detrusor compliance
Inosine [93]	antioxidant by peroxynitrite disattivation, anti-inflammatory, axogenic and neurotrophic properties	Modulation of detrusor overactivity, decrease of non-voiding contraction (NVC), decrease TRPV1 in bladder tissue	Intraperitoneal injection in rat with NVC immediately after SCI
Mirabegron [94]	β-3 agonist	Urodynamic improvement	Administered in patients with neurogenic detrusor overactivity (NDO) after SCI
Naftopidil/BMY7378/Silodosin (α-adrenoceptor blockers) [95]	α-adrenoceptor blockade	Reduction of urethral resistance, voiding efficiency improvement by external urethral sphincter-electromyography(EMG)	Intravenous injection in rat with chronic SCI
Propiverine (antimuscarinic agent) [96]	Antagonism against muscarinic receptor, L-type Ca^2+^ channels and transient receptor potential vanilloid subtype 1 (TRPV1)	Amelioration of urinary tract dysfunctions and reduction of detrusor overactivity	Administered to rats with SCI and non-voiding contraction (NVC)

**Table 6:** Materials employed for production of nanofibers proposed for SCI treatment.

Materials Employed	Drug Loaded	Potential Effect in SCI
Ac-FAQ with PCL+ PLGA [110]	-	In vivo nerve regeneration
*Bombyx mori* silk fibroin (SF) [111]	-	In vitro neurite outgrowth and astrocyte migration
Chitosan scaffold [112]	-	In vivo functional recovery
Collagen type I [113]		In vivo neurite outgrowth and astrocyte migration
Collagen type I [114]	-	In vivo motor recovery
Graphene nanoscaffold [115]	-	In vivo biocompatibility and nerve outgrow
Multi-layer PCL [116]	-	In vitro axonal regeneration
PCL + Gum tragacanth (GT) [117]	Curcumin	In vitro biocompatibility, long-lasting release of drug and wound healing properties
Peptide anphiphile (PA) [118]	Dexamethasone	Achievement of long-lasting release of drug and In vivo localized anti-inflammatory effect
PCL [119]	Dexamethasone	Achievement of long-lasting release of drug
PCL + PLGA functionalized with Ac-FAQ [110]	-	In vivo nerve regeneration
PLA [120]	-	In vivo biocompatibility and promotion of spinal cord damage repair
PLGA + PCL + (RADA16, a ionic self-complementary peptide) [121]	Cytokines	In vivo axonal regeneration and neurological recovery
PLGA [98]	-	In vivo axonal regeneration and motor and sensory recovery
PLA + gum tragacanth (PLA/GT) [117]	-	In vitro neurite outgrowth and nerve cell elongation on aligned nanofibers
PPC [60]	Dibutyryl cyclic adenosine monophosphate (dbcAMP)	In vivo nerve regeneration, functional recovery and glial scar reduction
Poly(trimethylene carbonate-co-ε-caprolactone) [122]	Ibuprofen	In vivo nerve conduit and anti-inflammatory
Positively charged oligo[poly(ethylene glycol)fumarate] (OPF+) [123]	-	In vivo axonal regeneration and functional recovery
PuraMatrix nanofibrous hydrogel + honeycomb collagen sponge [107]	-	In vivo locomotion functional recovery, spinal repair and neuronal regeneration
Electrospun PLGA coated with polypyrrole (PPy) [124]	-	Electrical stimulation and topographical guidance In vitro on PC12 cells improved neurite outgrowth
PCL/collagen/nonobioglass(NBG) [125]	-	Human Endometrial Stem cells adhesion and proliferation
(Ser-Ile-Lys-Val-Ala-Val)-modified poly(2-hydroxethyl methacrylate) (PHEMA) [126]	-	In vivo tissue bridging and aligned axonal ingrowth
Poly(glycerol sebacate) (PGS) + poly(methyl methacrylate) (MMA) with and without gelatin [127]		PC12 cells proliferation
Hyaluronic acid (HA) + PCL [128]		Attachment of SH-SY5Y neuroblastoma cells
SNF coated with poly-d-lysine (PDL) or (3-aminopropyl) trimethoxysilane (APTS) [129]	-	Promotion of In vitro neuron growth and neurite density increase
Tussah silk fibroin (TSF) [130]	-	In vitro improvement of olfactory ensheathing cell (OECs) neuro-regenerative potential
Gelatin (GL) + polyethylene-oxide (PEO) + (3-Glycidoxypropyl) methyldiethoxysilane(GPTMS) [131]		Schwann cells proliferation
PCL-Chitosan [132]	Laminin	Schwann cells grown

**Table 7:** Self-assembling peptides reported in the literature for treatment of SCI.

Self-Assembling Peptides	Animal Model	Potential Effect in SCI
Biotin B24 (GGGAFASTKT-CONH2) [166]	Murine contusion model	Low infiltration of CD68 + macrophages and iba + microglia
Biotin LDLK12 (LDLKLDLKLDLK-CONH2) [166]	Murine contusion model	Low infiltration of CD68 + macrophages and iba + microglia
Laminin epitope CQIK (Ac-(RADA)4GGCQAASIKVAV-CONH2) [167]	Motor recovery in SCI model	Higher neural differentiation of hEnSCs (human endometrial-derived stromal cells,) neurite outgrowth and myelination
Laminin epitope IKVAV-peptide amphiphile (PA) [163]	Murine spinal cord contusion and compression model	Promotion of functional recovery
SAP: K2(QL)6K2 [139]	Murine model clip compression	Improvement of locomotion function attenuation of inflammation
